# Analysis of Risk Factors for Surgical Site Infection in Obstetric Surgeries at a Tertiary Care Hospital in South India: A Cross-Sectional Study

**DOI:** 10.7759/cureus.95063

**Published:** 2025-10-21

**Authors:** Saru Sree, Logeswari B M, Abirami V

**Affiliations:** 1 Obstetrics and Gynaecology, Sree Balaji Medical College and Hospital, Chennai, IND

**Keywords:** antibiotic sensitivity pattern, caesarean section, medical comorbidities, risk factors, surgical site infection(ssi)

## Abstract

Background: Surgical site infections (SSIs) are among the most common postoperative complications, contributing significantly to maternal morbidity and healthcare burden worldwide. Obstetric procedures, particularly cesarean sections, are prone to SSIs due to exposure to vaginal and enteric flora, patient comorbidities, and procedural factors. This study aimed to assess the incidence, risk factors, comorbidities, and microbiological characteristics of SSIs in obstetric surgical patients.

Methods: An observational, cross-sectional study was conducted over 12 months in the Department of Obstetrics and Gynaecology at Sree Balaji Medical College and Hospital, Chennai, India. A total of 300 women undergoing obstetric surgery were enrolled using a convenience sampling technique. Detailed demographic, clinical, and surgical data were collected. SSIs were defined according to the Centers for Disease Control and Prevention (CDC) and National Healthcare Safety Network (NHSN) criteria as infections occurring within 30 days postoperatively. Wound swabs from suspected SSIs were cultured, and antimicrobial susceptibility was determined. Statistical analysis was performed using chi-square tests, with p < 0.05 considered significant.

Results: The overall incidence of SSIs was 20/300 (6.7%). Infection rates were higher in emergency surgeries, 16/222 (7.2%), compared to elective procedures, 4/78 (5.1%). Significant risk factors included age 30-40 years 10/80 (12.5%, p = 0.020), obesity 12/50 (24%, p = 0.001), diabetes mellitus 12/50 (24%, p < 0.001), preoperative vaginal discharge 7/23 (30.4%, p = 0.001), multiple vaginal examinations (>5) 12/50 (24%, p = 0.001), prolonged hospital stay (>14 days) 5/30 (16.7%, p = 0.008), and use of interrupted sutures 12/100 (12%, p = 0.018). The predominant microbial isolates were *Escherichia coli* 5/20 (25%) and MRSA 4/20 (20%), with 7/20 (35%) of cases showing no growth. Linezolid, amikacin, and gentamicin demonstrated the highest susceptibility.

Conclusion: SSIs remain a significant complication of obstetric surgery, particularly in high-risk patients and emergency settings. Optimizing maternal comorbidities, adhering to aseptic techniques, judicious antibiotic use, and implementing robust infection control strategies are essential to reduce SSI incidence and improve maternal outcomes.

## Introduction

Surgical site infections (SSIs) remain a prominent category of healthcare-associated infections, posing significant challenges to maternal health, particularly in obstetrical care settings. SSIs contribute substantially to postoperative morbidity, with cesarean deliveries (CDs) representing a key area of concern due to their increasing frequency and associated complications. In India, the rising rate of CDs has amplified the potential burden of SSIs. A global meta-analysis encompassing 43 studies from 29 countries, involving over 798,000 patients, estimated the pooled incidence of SSIs at 2.5% (95% Confidence Interval: 1.6-3.7%) [1], underscoring the worldwide relevance of this problem. Concurrently, the National Family Health Survey-5 (NFHS-5) reported a national CD rate exceeding 21%, with certain private healthcare facilities documenting rates above 50% [2]. This rising trend in CDs reflects a complex interplay of medico-legal considerations, societal expectations, and evolving clinical practices, alongside an increasing prevalence of maternal comorbidities, including obesity, diabetes mellitus, and hypertension.

SSIs following cesarean deliveries can range from superficial incisional infections to deeper organ or space infections, each carrying distinct clinical implications. Multiple patient-related factors have been identified that elevate the risk of postoperative infection. High body mass index (BMI), metabolic disorders, prolonged labor, multiple vaginal examinations, premature rupture of membranes (PROM), and emergency surgical procedures are all recognized contributors to increased SSI risk. In addition, anemia, a condition with high prevalence among pregnant individuals in India, impairs tissue oxygenation and healing, further predisposing women to postoperative infections [3,4].

Beyond individual patient factors, structural and procedural inadequacies within healthcare facilities exacerbate the risk of SSIs. Overcrowded surgical units, lapses in aseptic technique, and suboptimal infection prevention and control (IPC) protocols contribute significantly to infection rates. Inconsistent adherence to established guidelines for prophylactic antimicrobial use, as recommended by the World Health Organization (WHO) and the Indian Council of Medical Research (ICMR), undermines efforts to prevent SSIs. Moreover, practices such as the reuse of surgical instruments, insufficient hand hygiene compliance, and the indiscriminate use of broad-spectrum antibiotics have facilitated the emergence and dissemination of multidrug-resistant pathogens, including methicillin-resistant *Staphylococcus aureus* (MRSA) and extended-spectrum beta-lactamase (ESBL)-producing organisms [3-5].

Effective prevention of SSIs in obstetrical surgery requires a comprehensive, multi-dimensional approach. Evidence-based frameworks, such as the Robson Classification, can guide judicious decision-making regarding the need for cesarean delivery, thereby reducing unnecessary surgical interventions. Prenatal care programs should focus on modifiable risk factors by screening and treating anemia, encouraging appropriate gestational weight gain, and identifying infections early. Within healthcare facilities, the standardization of IPC protocols, routine monitoring through audits, and ongoing training of healthcare personnel are critical components of SSI prevention, alongside timely administration of prophylactic antibiotics [5,6].

Hospital-based surveillance systems for SSIs, coupled with microbiological monitoring and the establishment of active institutional infection control committees, enable early detection and effective management of postoperative infections. In addition, patient education initiatives aimed at recognizing early signs of infection and adopting hygienic practices play a pivotal role in reducing SSI incidence [7-9]. Despite growing research efforts on SSIs following cesarean deliveries, several knowledge gaps persist, limiting the implementation of universally applicable preventive strategies. Studies by Arora et al. highlight discrepancies between recommended guidelines and real-world practice, revealing suboptimal adherence to protocols even in tertiary care settings [6]. Addressing these gaps necessitates targeted investigation into the risk determinants of SSIs, coupled with the development and implementation of standardized preventive measures.

In conclusion, reducing SSIs in obstetrical surgery requires an integrated approach that encompasses patient-centered interventions, structural improvements within healthcare facilities, and behavioral modifications among healthcare providers. Focused research on risk factors and consistent application of evidence-based prevention strategies are essential to improving maternal outcomes and mitigating the spread of antimicrobial resistance in India. This study aimed to measure the incidence of surgical site infection in obstetric surgeries and also to evaluate the risk factors for surgical site infection in patients undergoing surgery at the Department of Obstetrics and Gynaecology.

## Materials and methods

Study design

This research was designed as a prospective, observational, cross-sectional study aimed at evaluating the risk factors for surgical site infections (SSIs) in obstetric surgeries. By adopting a prospective approach, the study allowed for real-time monitoring of patients from the point of surgical intervention through the postoperative period, thereby enabling accurate capture of events as they occurred. The observational nature of the study ensured that patients were managed according to routine clinical protocols without any experimental manipulation, maintaining ethical and practical feasibility in a busy obstetric setting. Cross-sectional evaluation provided a snapshot of SSI occurrence and associated risk factors within the study population, permitting the identification of correlations between patient characteristics, surgical parameters, and infection outcomes.

Study duration and setting

The study was conducted over a continuous 12-month period, providing a comprehensive assessment of SSIs across various seasons and patient influxes, which could influence infection patterns. The research was undertaken in the Department of Obstetrics and Gynaecology at Sree Balaji Medical College and Hospital (SBMCH), located in Chennai, Tamil Nadu, India. This tertiary care institution serves a diverse urban and semi-urban population, offering both elective and emergency obstetric surgical services. The hospital's well-equipped surgical and infection control facilities allowed for standardized monitoring and consistent data collection, ensuring that all relevant perioperative factors could be systematically assessed.

Study population and sampling

The study population comprised women undergoing obstetric surgical procedures, including both elective and emergency cesarean sections, admitted to the obstetric unit during the study period. A non-probability convenience sampling technique was employed, whereby all eligible patients presenting during the study timeframe were considered for inclusion. This method was chosen due to its feasibility in capturing a representative cohort within the constraints of clinical workflow and hospital admission patterns. Inclusion criteria mandated that participants be women undergoing obstetric surgery at SBMCH during the defined period and willing to provide informed written consent. Exclusion criteria eliminated patients with pre-existing local or systemic infections at the time of surgery, those who declined consent, and individuals lost to follow-up before evaluation of wound healing status. By carefully defining inclusion and exclusion parameters, the study sought to focus specifically on infections arising as a direct consequence of obstetric surgical interventions rather than pre-existing conditions.

Sample size determination

The required sample size was estimated using Dobson’s formula for determining a single proportion in a cross-sectional study. In this formula, nn represents the minimum required sample size, Z denotes the standard normal deviate corresponding to a 95% confidence level (1.96), p signifies the anticipated prevalence of SSIs, and dd reflects the absolute precision or margin of error. Based on preliminary hospital data and published literature [10], the prevalence of SSIs following obstetric surgery was estimated at 6.7%. Setting the margin of error at 5% (d = 0.05), the calculation yielded a minimum sample size of 96 participants. To account for potential attrition, loss to follow-up, and incomplete data, a 10% non-response adjustment was incorporated, resulting in an adjusted minimum sample size of 107. Nevertheless, to enhance statistical power and allow for meaningful subgroup analyses, the study ultimately enrolled 300 obstetric surgical cases, ensuring robust and reliable results.

Data collection

Comprehensive patient information was collected using a structured proforma designed to capture demographic characteristics, relevant medical history, surgical details, and postoperative monitoring parameters. Demographic data included age, parity, body mass index (BMI), socioeconomic status, and comorbidities such as diabetes mellitus, hypertension, and anemia. Surgical details encompassed the type of procedure (elective versus emergency), duration of surgery, estimated blood loss, number of vaginal examinations prior to surgery, membrane status, and intraoperative complications. Postoperative monitoring focused on wound assessment, clinical signs of infection, and any interventions performed. The use of a standardized proforma ensured uniformity in data collection across different patients and reduced observer bias.

Definition and assessment of surgical site infection

SSIs were defined according to the Centers for Disease Control and Prevention (CDC) and National Healthcare Safety Network (NHSN) guidelines. An SSI was considered present if any infection developed within 30 days of surgery, affecting the skin, subcutaneous tissue, fascia, muscle, or deeper structures at the surgical site [7]. Diagnosis of SSI was based on the presence of purulent discharge from the wound, localized signs such as pain, redness, warmth, or swelling, or a clinician’s assessment indicating infection based on wound evaluation and clinical suspicion. By adhering to standardized criteria, the study ensured objective classification of infection outcomes and facilitated comparability with other studies. In this study, both superficial and deep incisional surgical site infections were included, as per CDC/NHSN guidelines.

Microbiological analysis

In cases where an SSI was suspected, wound exudate or pus samples were collected under sterile conditions and sent to the microbiology laboratory for culture and sensitivity testing. Microorganisms were isolated using established bacteriological methods, and antimicrobial susceptibility testing was conducted according to Clinical and Laboratory Standards Institute (CLSI) guidelines. This microbiological evaluation allowed for the identification of causative pathogens and determination of resistance patterns, which is critical for both patient management and broader infection control efforts within the hospital.

Statistical analysis

Data were systematically entered and managed using Microsoft Excel to ensure accurate record-keeping and facilitate subsequent analysis. Statistical analyses were performed using Statistical analysis was performed using IBM Corp. Released 2019. IBM SPSS Statistics for Windows, Version 25. Armonk, NY: IBM Corp. Descriptive statistics, including means, standard deviations, and proportions, were used to summarize demographic characteristics, comorbidities, surgical details, and SSI outcomes. The chi-square test (χ²) was employed to assess the association between categorical risk factors and the occurrence of SSIs. A p-value of <0.05 was considered statistically significant, indicating a meaningful relationship between variables. This analytical approach allowed the identification of significant predictors of SSIs and provided evidence for targeted interventions.

Ethical considerations

The study was conducted in strict compliance with the ethical principles outlined in the Declaration of Helsinki. Informed written consent was obtained from all participants, who were assured that participation was voluntary and that withdrawal at any stage would not affect their medical care. Ethical approval was granted by the Institutional Human Ethics Committee of Sree Balaji Medical College (Approval No. 002/SBMCH/IHEC/2023/2041). Patient confidentiality was maintained at all stages, and data were anonymized for analysis. Ethical safeguards ensured that the study met international standards for human subject research while maintaining patient safety and rights.

## Results

**Figure 1:**
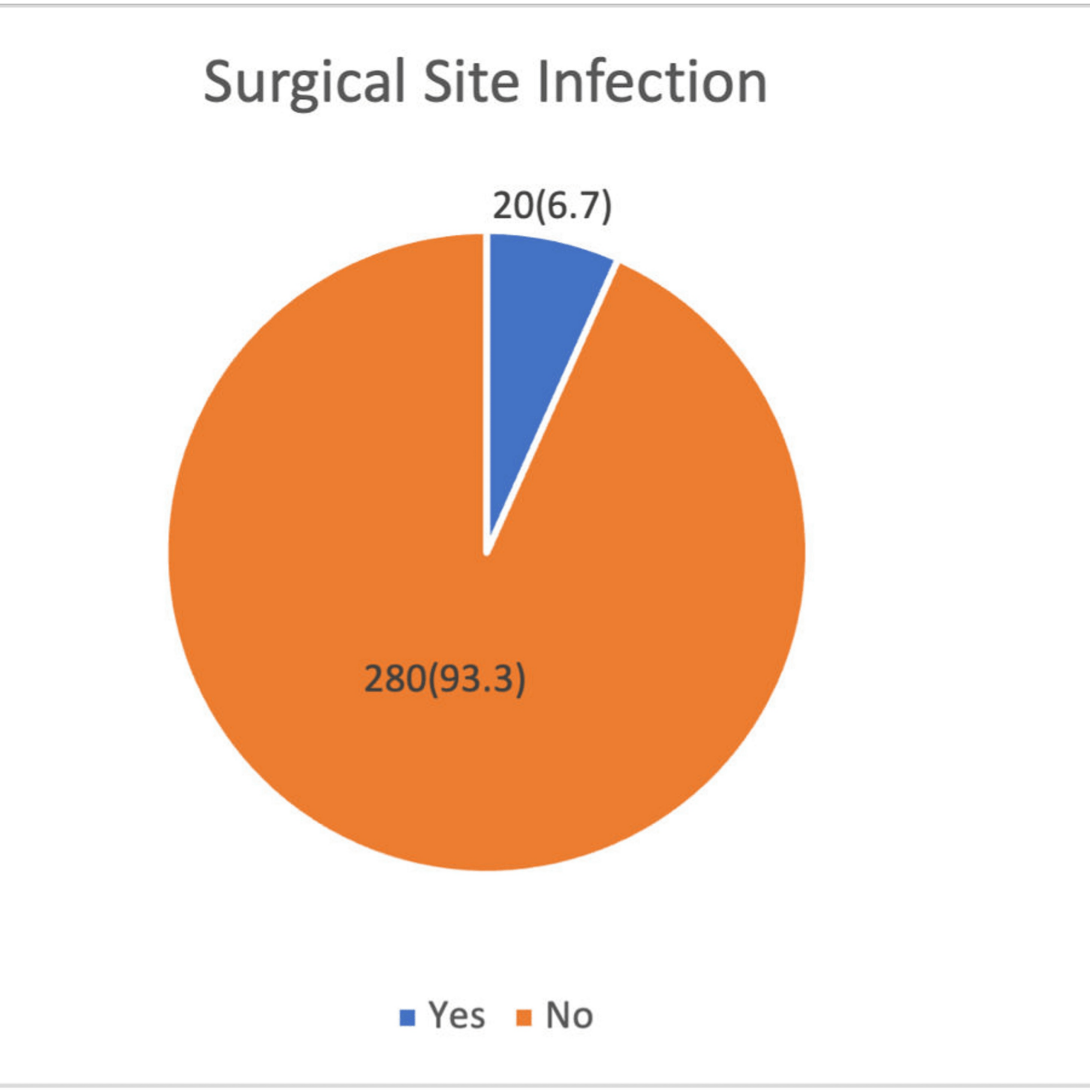
Incidence of surgical site infection (SSI) among obstetric surgery cases in the study population (n=300)

Figure 1 illustrates the incidence of surgical site infection (SSI) among obstetric surgery cases, showing that out of a total of 300 procedures, 20 cases (6.7%) experienced SSIs, while the vast majority, 280 cases (93.3%), did not develop infection. This indicates that surgical site infections remain relatively infrequent in this cohort, reflecting effective perioperative care and infection control practices in the obstetric surgical population.

Table 1 shows the association of demographic, clinical, and surgical factors with surgical site infections (SSIs) among 300 obstetric surgery patients. The overall incidence of SSI was 20 cases (6.7%), while 280 women (93.3%) did not develop infection. The type of surgery was not significantly associated with infection, with 4 out of 78 elective procedures (5.1%) and 16 out of 222 emergency procedures (7.2%) developing SSI (χ² = 0.37, p = 0.541). Age showed a significant relationship (χ² = 7.85, p = 0.020), with SSIs occurring in 2/100 women <20 years (2.0%), 8/120 women aged 20-30 years (6.7%), and 10/80 women aged 30-40 years (12.5%). Parity did not influence infection risk (χ² = 0.20, p = 0.650), with 2/50 primigravida (4.0%) and 18/250 multiparous women (7.2%) developing SSI. Body mass index (BMI) was strongly associated with infection (χ² = 23.76, p = 0.001). Among women with normal BMI, only 2/150 (1.3%) developed SSI, compared with 6/100 overweight women (6.0%) and 12/50 obese women (24.0%). Comorbidities also showed a significant association (χ² = 21.12, p = 0.001). The highest SSI incidence was among women with diabetes mellitus (12/50, 24.0%), followed by those with other comorbidities (2/22, 9.1%), hypertension (3/79, 3.8%), and anemia (3/149, 2.0%).

**Table 1:** Association of demographic, clinical, and surgical factors with surgical site infections (SSI) among obstetric surgery patients (N = 300) * Chi-square/Fischer’s exact test, p-value <0.05 considered statistically significant, # Asthma, CKD: Chronic kidney disease, Hypothyroidism, CHD: Coronary heart disease

Factor	Category	SSI Yes (n, %)	SSI No (n, %)	χ² value	p-value
Type of Surgery	Elective	4 (5.1%)	74 (94.8%)	0.37	0.541
	Emergency	16 (7.2%)	206 (92.8%)		
Total		20 (6.7%)	280 (93.3%)		
Age (years)	<20	2 (2.0%)	98 (98.0%)	7.85	0.020*
	20–30	8 (6.7%)	112 (93.3%)		
	30–40	10 (12.5%)	70 (87.5%)		
Parity	Primigravida	2 (4.0%)	48 (96.0%)	0.20	0.650
	Multiparous	18 (7.2%)	232 (92.8%)		
BMI	Normal	2 (1.3%)	148 (98.7%)	23.76	0.001*
	Overweight	6 (6.0%)	94 (94.0%)		
	Obese	12 (24.0%)	38 (76.0%)		
Comorbidities	Anemia	3 (2.0%)	146 (97.9%)	21.12	0.001*
	Hypertension	3 (3.8%)	76 (96.2%)		
	Diabetes Mellitus	12 (24.0%)	38 (76.0%)		
	Others#	2 (9.1%)	20 (90.9%)		

Table 2 shows the association of obstetric risk factors with SSI. Among women with a history of previous lower segment cesarean section (LSCS), SSI occurred in 2/80 (2.5%) (p=0.034). Other risk factors, such as prolonged labor (2/50, 4.0%), malpresentation (1/30, 3.3%), antepartum hemorrhage (1/20, 5.0%), preterm premature rupture of the membranes (PPROM) (8/71, 11.3%), and meconium-stained liquor (6/49, 12.2%), were associated with varying infection rates, though not all reached significance. Notably, vaginal examinations were strongly associated with SSI, with risk rising from 2.0% (2/100) in women without exams to 4.0% (6/150) with ≤5 exams and 24.0% (12/50) with >5 exams (χ² = 21.88, p = 0.001). Preoperative vaginal discharge was another significant predictor, with infection in 7/23 (30.4%) compared to 13/277 (4.7%) without discharge (χ² = 23.41, p = 0.001).

**Table 2:** Association of obstetric risk factors with surgical site infections (SSI) among obstetric surgery patients (N = 300) * Chi-square/Fischer exact test, a p-value <0.05 considered statistically significant LSCS: lower segment cesarean section, PPROM: premature rupture of the membranes

Factor	Category/Level	SSI Yes (n, %)	SSI No (n, %)	χ² value	p-value
Risk factors					
Previous LSCS	Yes (n=80)	2 (2.5%)	78 (97.5%)	4.49	0.034*
Prolonged labour	Yes (n=50)	2 (4.0%)	48 (96.0%)		
Malpresentation	Yes (n=30)	1 (3.3%)	29 (96.7%)		
Antepartum hemorrhage	Yes (n=20)	1 (5.0%)	19 (95.0%)		
PPROM	Yes (n=71)	8 (11.3%)	63 (88.7%)		
Meconium-stained liquor	Yes (n=49)	6 (12.2%)	43 (87.8%)		
Vaginal Examinations	None (n=100)	2 (2.0%)	98 (98.0%)	21.88	0.001*
	≤5 (n=150)	6 (4.0%)	144 (96.0%)		
	>5 (n=50)	12 (24.0%)	38 (76.0%)		
Pre-op Vaginal Discharge	Absent (n=277)	13 (4.7%)	264 (95.3%)	23.41	0.001*
	Present (n=23)	7 (30.4%)	16 (69.6%)		

Table 3 presents perioperative and postoperative risk factors for SSI. Although longer surgical duration (>1 hour) showed higher infection (18/232, 7.8%) compared to shorter procedures (2/68, 2.9%), the difference was not significant (p=0.160). The type of anesthesia was significant, with general anesthesia linked to a higher SSI rate (10/70, 14.3%) than local/regional anesthesia (10/230, 4.3%) (χ² = 8.23, p = 0.004). Prophylactic antibiotic choice also influenced SSI, with cefotaxime showing the highest infection rate (14/104, 13.4%) compared to ceftriaxone (4/152, 2.6%) and piperacillin-tazobactam (2/44, 4.5%) (χ² = 12.43, p = 0.002).

**Table 3:** Association of perioperative and postoperative risk factors with surgical site infections (SSI) among obstetric surgery patients (N = 300) * Chi-square/Fischer’s exact test, a p-value <0.05 considered statistically significant

Factor	Category/Level	SSI Yes (n, %)	SSI No (n, %)	χ² value	p-value
Surgery Duration	≤1 hour (n=68)	2 (2.9%)	66 (97.1%)	1.97	0.160
	>1 hour (n=232)	18 (7.8%)	214 (92.2%)		
Type of Anesthesia	Local/Regional (n=230)	10 (4.3%)	220 (95.7%)	8.23	0.004*
	General (n=70)	10 (14.3%)	60 (85.7%)		
Antibiotic Used	Cefotaxime (n=104)	14 (13.4%)	90 (86.6%)	12.43	0.002*
	Ceftriaxone (n=152)	4 (2.6%)	148 (97.4%)		
	Piperacillin-Tazobactam (n=44)	2 (4.5%)	42 (95.5%)		
Blood Transfusion	No (n=278)	18 (6.5%)	260 (93.5%)	7.85	0.005*
	Yes (n=22)	2 (9.1%)	20 (90.9%)		
Post-op Hospital Stay	1–7 days (n=180)	6 (3.3%)	174 (96.7%)	9.70	0.008*
	7–14 days (n=90)	9 (10.0%)	81 (90.0%)		
	>14 days (n=30)	5 (16.7%)	25 (83.3%)		
Suture Type	Subcuticular (n=200)	8 (4.0%)	192 (96.0%)	5.62	0.018*
	Interrupted (n=100)	12 (12.0%)	88 (88.0%)		
Suture Material	Prolene (n=170)	5 (2.9%)	165 (97.1%)	8.84	0.012*
	Ethilon (n=120)	14 (11.7%)	106 (88.3%)		
	Monocryl (n=10)	1 (10.0%)	9 (90.0%)		

Blood transfusion recipients had a higher SSI risk (2/22, 9.1%) compared to those who did not (18/278, 6.5%) (χ² = 7.85, p = 0.005). Postoperative hospital stay was strongly associated with SSI, increasing progressively from 3.3% (6/180) for ≤7 days to 10.0% (9/90) for 7-14 days, and 16.7% (5/30) for >14 days (χ² = 9.70, p = 0.008). Suture technique also played a role, with interrupted sutures showing higher infections (12/100, 12.0%) than subcuticular closure (8/200, 4.0%) (χ² = 5.62, p = 0.018). Similarly, Ethilon (14/120, 11.7%) and Monocryl (1/10, 10.0%) were associated with higher infection rates compared to Prolene (5/170, 2.9%) (χ² = 8.84, p = 0.012).

Table 4 presents the microbiological spectrum of SSIs and their antibiotic susceptibility pattern. Among culture-positive cases, *Escherichia coli* (5/20; 25%) was the most common isolate, followed by MRSA (4/20; 20%), *Klebsiella spp.* (3/20; 15%), and *Staphylococcus aureus* (1/20; 5%), while 7/20 (35%) of SSI cases showed no growth. The association between organism type and SSI occurrence was statistically significant (p < 0.001). Antibiotic susceptibility testing showed varied patterns: Linezolid (30%) and Amikacin (25%) demonstrated relatively higher sensitivity among isolates, while Ceftriaxone (10%), Gentamicin (15%), and Piperacillin-Tazobactam (20%) showed moderate activity. 

**Table 4:** Distribution of microorganisms isolated from surgical site infections (SSI) and their antibiotic susceptibility pattern (N = 300) * Chi-square/Fischer exact test A p-value <0.05 is considered statistically significant

Microorganism	SSI Yes n (%)	SSI No n (%)	Common Sensitive Antibiotics (n, %)	χ² value	p-value
Staphylococcus aureus	1 (5.0)	2 (0.7)	Ceftriaxone: 2 (10.0)	45.1	<0.001*
Klebsiella spp.	3 (15.0)	4 (1.4)	Amikacin: 5 (25.0)		
Methicillin-resistant Staphylococcus aureus (MRSA)	4 (20.0)	6 (2.1)	Gentamicin: 3 (15.0)		
Escherichia coli	5 (25.0)	8 (2.9)	Linezolid: 6 (30.0)		
No organism isolated	7 (35.0)	260 (92.9)	Piperacillin–Tazobactam: 4 (20.0)		

## Discussion

Obstetric surgical procedures inherently possess a diverse risk profile because they often occur in emergency settings where time constraints may limit preoperative optimization. Furthermore, the genital tract naturally harbors a wide array of microorganisms, including vaginal and enteric flora, that can contaminate surgical wounds. This accounts for the higher infection risk observed in obstetric procedures compared with clean surgical procedures such as abdominal hysterectomy [11]. Cesarean sections, the most common major obstetric surgery globally, are categorized as clean-contaminated procedures, reflecting the elevated risk associated with exposure to endogenous vaginal and gastrointestinal flora. Despite advances in aseptic techniques, the routine administration of prophylactic antibiotics, and improvements in surgical practices, the burden of SSI in obstetrics remains considerable, particularly in resource-limited healthcare settings [12].

The etiology of SSIs is multifactorial, with both patient-related and procedure-related risk factors contributing significantly to outcomes. In our study, several key obstetric and host-related variables were found to influence infection rates. Obstetric-specific risk factors, such as prolonged labor, premature rupture of membranes, and meconium-stained amniotic fluid, are well-established contributors to postoperative infection [13]. Prolonged labor and premature rupture of membranes increase the duration of microbial exposure before surgery, thereby facilitating ascending infection and seeding of the surgical wound. Similarly, meconium-stained liquor is a marker of fetal distress but also represents a risk for bacterial contamination. These findings underscore the importance of timely decision-making in labor management and surgical intervention to mitigate SSI risk. Although the difference in SSI rates between elective and emergency obstetric surgeries was not statistically significant, this finding may reflect the consistent implementation of standardized infection prevention and aseptic measures across all procedures. At our institution, adherence to uniform surgical safety checklists, antibiotic prophylaxis protocols, and postoperative wound care practices likely minimizes variation in infection risk between elective and emergency cases. Additionally, the relatively small number of SSI events could have limited the statistical power to detect subtle differences.

Host-related factors, including diabetes mellitus, anemia, obesity, and immunosuppressive conditions, significantly impair wound healing. Hyperglycemia, in particular, alters leukocyte function, reduces chemotaxis, and interferes with collagen synthesis, all of which contribute to impaired tissue repair and increased susceptibility to infection [14]. Anemia, by limiting tissue oxygenation, reduces the capacity for oxidative killing by neutrophils, further predisposing to infection. Obesity presents an additional risk by increasing operative time, impairing wound closure, and creating larger avascular tissue planes that are prone to infection. Together, these host factors highlight the importance of optimizing maternal health status both before and during pregnancy, as comorbidities substantially influence postoperative outcomes. Intraoperative factors also play a role in SSI development. The duration of surgery, the type of incision, intraoperative blood loss, and the choice of anesthesia have all been shown to influence infection outcomes [15]. Prolonged operative time, for instance, increases the opportunity for bacterial contamination. In emergency surgeries, the rapidity of decision-making and compromised aseptic protocols may further elevate risk.

The microbial landscape of SSIs in obstetric procedures is typically polymicrobial. In our cohort, the predominant isolates were *Escherichia coli*, methicillin-resistant *Staphylococcus aureus* (MRSA), and *Klebsiella pneumoniae*. These findings are consistent with global literature, where Gram-negative bacilli and drug-resistant Gram-positive organisms frequently predominate [16,7]. The recovery of MRSA in a significant proportion of cases is of particular concern, as it reflects not only hospital-acquired transmission but also the increasing prevalence of antimicrobial resistance within obstetric populations. The isolation of *E. coli* and *Klebsiella* also highlights the contribution of endogenous gastrointestinal and urogenital flora to infection. Alarmingly, 35% of cultures yielded no bacterial growth. This could be attributed to several factors, including the widespread practice of administering antibiotics prior to wound swab collection, suboptimal sample collection techniques, or the inability to culture anaerobic organisms using standard microbiological methods. Similar findings were reported by Mangram et al., who noted that culture-negative SSIs can significantly underestimate the true microbial diversity [18]. Improving diagnostic sensitivity, particularly with the use of anaerobic cultures and molecular methods, may help overcome this limitation. 

In terms of antimicrobial susceptibility, the isolates demonstrated high sensitivity to linezolid, amikacin, and gentamicin, while lower sensitivity was observed for ceftriaxone and piperacillin-tazobactam. This aligns with global trends where aminoglycosides and oxazolidinones remain reliable options against multidrug-resistant Gram-positive and Gram-negative pathogens. However, the frequent use of broad-spectrum antibiotics raises concerns about accelerating resistance patterns. These findings emphasize the need for targeted antimicrobial therapy guided by culture results, rather than empirical overuse, as part of robust antimicrobial stewardship programs. By tailoring antibiotic regimens to local sensitivity patterns, healthcare institutions can both improve patient outcomes and mitigate resistance development.

Our study found a significantly higher rate of infection among patients undergoing emergency surgeries (7.2%) compared to elective ones (5.1%). This disparity can be attributed to several factors, including limited opportunities for preoperative optimization, higher prevalence of obstetric complications necessitating emergency intervention, and greater procedural complexity. Additionally, the urgency of emergency procedures often compromises strict adherence to aseptic practices. These findings are in line with the observations of Acharya et al., who also documented higher SSI rates in emergent cesarean deliveries [19]. Thus, efforts to reduce unnecessary emergency procedures, enhance preoperative preparedness, and streamline surgical protocols may help reduce infection rates.

Patients aged 30-40 years in our study were found to have a significantly higher risk of SSI (12.5%, p = 0.020). This age group often represents multiparous women who are more likely to present with obstetric complications such as placenta previa, gestational diabetes, or hypertensive disorders of pregnancy [20]. Moreover, advanced maternal age is itself associated with increased surgical risk due to reduced physiological reserve. Among comorbidities, diabetes mellitus stood out as a particularly strong risk factor, with an infection rate of 24% and a highly significant association (p < 0.001). This finding corroborates earlier work by Slekovec et al., who demonstrated that poor glycemic control substantially increases postoperative morbidity [21]. Hypertension and anemia were also identified as important contributors. These associations highlight the need for meticulous preoperative risk stratification and targeted interventions such as strict glycemic control, correction of anemia, and management of hypertension prior to surgical intervention.

Prolonged postoperative hospitalization was strongly correlated with SSI occurrence (p = 0.008). This finding may reflect a bidirectional relationship, wherein extended hospital stays predispose to nosocomial colonization and infection, while infection itself necessitates longer hospitalization for intravenous antibiotic therapy and wound care. Kvalvik et al. similarly described hospital stay as both a risk factor for and a consequence of SSI [22]. From a health system perspective, this finding underscores the urgent need to reduce hospital-acquired infections through improved infection control practices and early mobilization protocols.

Limitations

Our study has several limitations. First, the sample size, though adequate to establish significant associations, was limited to a single tertiary care teaching hospital, which may restrict the generalizability of findings to other healthcare settings. Second, culture-negative infections accounted for a significant proportion of cases, potentially underestimating the true microbial spectrum due to prior antibiotic exposure or the lack of anaerobic culture techniques. Third, long-term maternal outcomes, such as chronic wound complications or recurrent infections, were not assessed. Convenience sampling was employed due to feasibility constraints and the availability of study participants during the data collection period. However, this method may introduce selection bias, as the sample may not accurately represent all obstetric patients undergoing surgery at the institution or in similar settings. Consequently, the findings may have limited external validity and may not be generalizable. Finally, molecular characterization of resistant strains, which could have provided deeper insights into antimicrobial resistance dynamics, was not undertaken due to resource constraints.

Implications and future directions

The findings of this study carry important clinical and public health implications. For clinicians, the strong association between comorbidities such as diabetes and the risk of SSI underscores the need for aggressive optimization of maternal health prior to surgery. For policymakers, the study highlights the pressing need to strengthen infection prevention and control programs in obstetric care, particularly in resource-constrained settings. Antimicrobial stewardship must be prioritized to preserve the efficacy of existing antibiotics, given the rising burden of multidrug-resistant organisms. Future research should focus on multicentric studies to enhance the generalizability of findings, employ advanced diagnostic methods, including anaerobic cultures and molecular assays, and evaluate long-term maternal and neonatal outcomes. By integrating clinical, microbiological, and public health approaches, a comprehensive strategy can be developed to mitigate the burden of SSI in obstetric populations. While our findings of MRSA, *E. coli*, and *Klebsiella pneumoniae* align with global trends, institutional antibiogram data or infection control surveillance reports would provide stronger support for these observations. However, due to the retrospective nature of the study, such data were not systematically available for correlation. Future studies incorporating local antibiogram trends and hospital surveillance data are warranted to better understand resistance patterns and guide empiric antibiotic policies for obstetric surgical site infections.

## Conclusions

Surgical site infections remain a significant challenge in obstetric practice, with multifactorial determinants spanning obstetric risk factors, host comorbidities, intraoperative variables, and microbial profiles. Our study demonstrated an overall incidence of 6.7%, with higher rates in emergency surgeries, patients aged 30-40 years, and those with comorbid conditions such as diabetes mellitus and anemia. The predominance of *Escherichia coli*, MRSA, and *Klebsiella* underscores the need for culture-directed therapy, while the high proportion of culture-negative cases highlights limitations in current diagnostic practices. Prolonged hospital stays both contributed to and resulted from SSI, amplifying the burden on healthcare resources. These findings emphasize the importance of preoperative optimization, strict adherence to aseptic techniques, rational antibiotic use, and strengthened infection control policies to reduce the incidence and impact of SSIs in obstetric populations.
